# The importance of review articles in making the voice of rare diseases heard: OJRD’s 10th anniversary

**DOI:** 10.1186/s13023-016-0456-5

**Published:** 2016-05-28

**Authors:** Ségolène Aymé

**Affiliations:** INSERM, US14, 96 rue Didot, 75014 Paris, France

“Join us in making the voice of rare diseases heard” is the slogan of Rare Disease Day 2016, a special day established by EURORDIS (http://www.rarediseaseday.org/). It takes place on the last day of February each year since 2008, to raise awareness amongst the general public and decision-makers about rare diseases and their impact on patients’ lives. This has also been one of the goals of *Orphanet Journal of Rare Diseases* (OJRD), since its inception 10 years ago.

This year’s slogan appeals to everyone who can make a difference but it appeals to us healthcare professionals and researchers first. We have a duty to create greater awareness for each of the thousands of rare diseases identified so far, by publishing the scarce data to which we have access.

*Orphanet Journal of Rare Diseases* (OJRD) is committed during this commemorative year and beyond, to publishing review articles on specific rare diseases with the goal of expanding knowledge among health professionals and caregivers. Such articles are highly valued by patients and caregivers, especially for very rare diseases. Summarising the available published literature is a major effort requiring experience, an asset of only a few professionals around the world. It is also a time-consuming exercise which is often postponed by many experts who are already overloaded. OJRD invites the rare disease community of experts to write up such reviews as the necessary step to make possible the dissemination of knowledge on diseases which are never taught at medical school. According to the Orphanet database, which systematically collects such articles provided that the copyright holders give permission to provide free access, there are currently 400 review articles published in peer-review journals, covering 1132 rare diseases. There are also 625 gene reviews documenting 1289 rare genetic diseases. Additional effort is needed to update these reviews and produce new ones.

Review articles are necessary and useful, but there is another type of compilation of scarce knowledge which can make an even more direct difference for the patients. It is the publication of clinical guidelines designed to support the decision-making process in patient care. This is the only way to champion improvements in quality and consistency in healthcare. The methodology for establishing such clinical guidelines is standardised in many countries by Health Technology Assessment (HTA) agencies. Clinical guidelines encode evidence-based recommendations, a feature hardly accessible in the case of most rare diseases as there are very few published articles reviewing the outcome of different protocols. Most clinical guidelines can only be defined due to the gathering of expert opinions which is considered the lowest possible level of evidence, but is still much better that nothing. The Orphanet database provides access to 100 clinical guidelines covering 470 rare diseases, a very low number indeed. They were produced either by learned societies, charities, by disease-specific consortiums, or HTA agencies. They are very costly to produce due to the intensity and complicated nature of the process that has to be respected. They have to ensure that they not only reflect the gathered evidence, but also fit within the healthcare systems as they define healthcare pathways. It is the reason why only a few of them can be considered to be “International” clinical guidelines, most of them being quite specific to a country or a region. OJRD is inviting funders to support the development of clinical guidelines and will support their publication.

For some rare diseases it is also indispensable to provide emergency guidelines for pre-hospital emergency care as well as for hospital emergency care, as some interventions can be decisive, negatively or positively, in the recovery of the patient. These are even more difficult to produce and translate into recommendations which can be widely applicable. Currently the Orphanet database gives access to 44 emergency guidelines for 85 rare diseases (http://www.orpha.net/consor/cgi-bin/Disease_Emergency.php?lng=EN). They were produced by French reference centres and patient organisations, edited by Orphanet, peer-reviewed by emergency practitioners, and then translated into English.

Patients suffering from a rare disease are more likely to need inpatient care, surgery and anaesthesia. Perioperative complications may occur in this setting, especially if the medical team lacks specific knowledge of the disease. Reliable information on orphan diseases is essential to avoid preventable disease-related complications such as unexpectedly difficult intubations, electrolytic imbalances, or adverse reactions or interactions of medication. The German Society of Anaesthesiology and Intensive Care developed OrphanAnaesthesia, (http://www.orphananesthesia.eu/en/home.html) an anaesthesiology-related database on orphan diseases which now contains over 30 guidelines published in German and English (written and peer reviewed by experts). Projects like these need more volunteers to contribute towards the production of potentially life-saving recommendations.

OJRD is also committed to contributing to the debate on appropriate policies for ensuring optimal care for patients and giving a voice to patients whose insight into the issues is a core asset. To this aim, OJRD and Findacure (http://www.findacure.org.uk/) joined efforts earlier this year to provide an opportunity for medical students to write an essay on a facet of rare diseases. One selected essay, which was published as a Letter to the Editor [1] in OJRD, is the clearest invitation to the rare disease community of experts to take the necessary time to disseminate their knowledge as soon as possible. OJRD intends to provide a forum for these experts and their patients’ voices, for decades to come.
